# The Role of Reduced Graphene Oxide in Enhancing the Mechanical and Thermal Properties of a Rubber Cover Joint

**DOI:** 10.3390/polym16081143

**Published:** 2024-04-18

**Authors:** Hongyu Zhang, Junxia Li, Wenrui Fan

**Affiliations:** 1 College of Mechanical and Vehicle Engineering, Taiyuan University of Technology, Taiyuan 030024, China; zhanghongyu0011@link.tyut.edu.cn (H.Z.); fanwenrui0018@link.tyut.edu.cn (W.F.); 2 Shanxi Province Engineer Technology Research Center for Mine Fluid Control, Taiyuan 030024, China; 3 National-Local Joint Engineering Laboratory of Mining Fluid Control, Taiyuan 030024, China

**Keywords:** conveyor belt, rubber cover joint, reduced graphene oxide, mechanical properties

## Abstract

The development of high-performance rubber composites has always been a research hotspot in the field of conveyor belt manufacturing. In this work, a rubber cover joint composite made of reduced graphene oxide (rGO) was prepared using latex mixing and mechanical blending methods, with a steel wire rope conveyor belt as the research object, and the influence of the rGO content on the properties of the rubber composite is discussed. The structure and morphology characterization of the rGO/NR rubber show that the addition of rGO does not change its crystal structure, and 1.2 phr rGO is uniformly dispersed throughout the rubber composite. As more rGO is added, the mechanical properties of the rGO rubber cover joint first improve and then worsen. With the addition of 1.2 phr, the cross-linking density increases by 80.6%, the tensile strength of the rubber composites increases by 49.7%, the elongation at break increases by 23.6%, and the adhesion strength increases by 12.4%. The tensile strength of the rGO rubber cover joint can still maintain 72.5% of its pre-thermal aging value. The wear resistance and thermal conductivity increase as more phr is added. When 3.0 phr is added, the wear resistance of the rubber composites increases by 32.9%, the thermal conductivity increases by 118.8%, and the temperature difference at the completion of vulcanization decreases from 4.5 °C to 1.8 °C. The results show that when 1.2 phr of rGO is added, the rubber conveyor belt joint obtains the best comprehensive performance. These enhanced comprehensive properties allow for the practical application of rGO nanomaterials to conveyor belt rubber.

## 1. Introduction

Steel cord conveyor belts are widely used in the logistic and transportation fields, such as the coal industry, mining, and metallurgy, due to their high tensile strength, high friction resistance, and strong impact resistance [1]. With the rapid development of long-distance and large-capacity conveyors, the requirements for conveyor belt performance are constantly changing [2]. A complete steel cord conveyor belt is made by splicing several short belts using vulcanization. Due to factors such as the quality of the rubber joint and the vulcanization process, the vulcanized joints remain the weakest point regardless of the quality of the conveyor belt as a whole.

**Figure 1:**
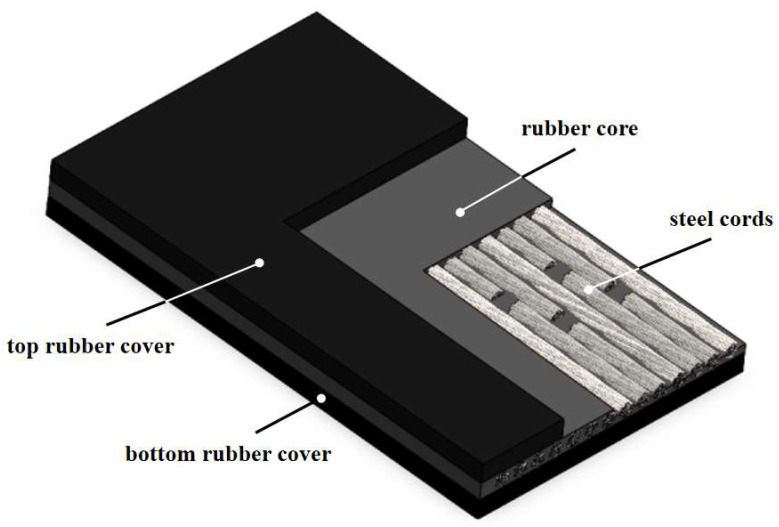
The joint of a steel wire rope core conveyor belt.

The structure of a steel cord core conveyor belt joint is shown in Figure 1. The top and bottom rubber covers encase a rubber core and steel cords arranged in a specific manner. The rubber cover, as the outermost layer of the steel cord core conveyor belt, comes into direct contact with the drums, idlers, and conveyed materials, protecting the rubber core and steel cords from the effects of wear, scraping, chemical corrosion, and other external environmental factors. It is the most vulnerable and consumable part of the entire conveyor belt, requiring the most maintenance and repair [3]. In the production of conveyor belt joints, a rubber cover with the same or better quality as the original rubber belt is typically used to achieve the best vulcanization effect. Therefore, improving the original conveyor belt formulation is an important approach to enhance the performance of the joints [4].

Scholars have strengthened rubber covers by adding fillers, such as white carbon black and natural fibers, achieving certain effects [5,6]. However, due to the introduction of other substances, the new rubber cover joint often experiences reduced adhesion strength with the original rubber cover and core. This can easily lead to negative consequences, such as the delamination and bulging of the conveyor belt. Carbon black, as an important additive in conveyor belt rubber, is used to improve its strength and wear resistance. Reduced graphene oxide (rGO), another carbon material similar to carbon black, possesses excellent electrical, thermal, mechanical, and other properties due to its single-atom-thick two-dimensional layer structure. For instance, rGO exhibits an ultra-high specific surface area (2630 m^2^/g) and outstanding physical mechanical strength. As a result, rGO is widely applied in the field of rubber composite materials [7,8,9]. In 2020, A.S. Sethulekshmi et al. [10] described the enhancement and application of graphene (GE), graphene oxide (GO), rGO, and their hybrids to natural rubber (NR). Compared to GO, rGO also provides the multifunctional enhancement of polymer nanocomposites. NR nanocomposites with improved mechanical and electrical properties can be obtained by inserting rGO into the NR matrix. Zhu et al. [11] investigated the enhanced mechanical properties, wet skid resistance, rolling resistance, heat build-up reduction, and wear resistance of RGO/SiO_2_-SSBR/BR composites, suggesting their potential application in green tires. Ma et al. [12] used rGO as a reinforcing material to prepare IIR/rGO composites with excellent mechanical properties using improved ultrasonic latex mixing and performing an in situ reduction process on the butyl latex (IIR) matrix. The results showed that the tensile strength and elongation at break of the composites increased together. Yan et al. [13] investigated the production and characterization of rGO/NR composites, revealing that the dispersion morphology of rGO significantly influences the chemical crosslink structure, mechanical properties, and barrier performance of the composites, highlighting the crucial role of filler morphology in determining the properties of natural rubber composites. In 2022, Cheng et al. [14], from North Central University, prepared rGO rubber materials with a balanced comprehensive performance by chemically modifying GO with gelatin and evaluated the temperature field distribution of rGO rubber tires using the finite element simulation method. The results showed that the composite material could effectively reduce the maximum temperature of the tires by 35%.

In recent years, the interaction between polymers and graphene-based substrates like rGO has been extensively explored using both experimental and theoretical/simulation approaches [15,16]. However, academic research on rGO rubber composite materials has mainly focused on tire manufacturing and single-rubber formulations. Conveyor belt cover joints, on the other hand, are composite materials composed of multiple rubbers and numerous fillers. Therefore, the aforementioned studies may not fully reflect the role and impact of rGO in actual conveyor belt joints. Additionally, there is scarce literature on the direct application of rGO to commercial conveyor belt products. Therefore, we chose the rubber cover joint, which is very important in the vulcanization operation of conveyor belt joints, as the material to explore the effect of rGO on the mechanical properties of the rubber cover joint in a commercial complete formula and the effect of rGO on the heat transfer of conveyor belts during vulcanization heating. This study is of great significance for improving the quality of conveyor belt joints and reducing belt failure accidents.

In this work, an ST 1600 steel wire rope conveyor belt produced by Aolun Belt Co., Ltd., (Yangquan, China) was taken as the research object. An rGO joint rubber cover was prepared using latex blending and mechanical blending methods based on its original commercial formula, and the influence of rGO on the mechanical properties of the rubber cover was studied. Finally, a vertical temperature-testing platform for conveyor belts was built to analyze the temperature transfer of the rGO rubber cover joint during the vulcanization process.

## 2. Experimental Section

### 2.1. Chemicals

rGO (G1000) was supplied by the Institute of Coal Chemistry, Chinese Academy of Sciences (Taiyuan, China); calcium chloride (CaCl_2_) and toluene were obtained from the China Beijing Chemical Plant (Beijing China); natural latex (NR, 60% dry rubber content), butadiene–styrene rubber (SBR), and all the vulcanization auxiliaries, i.e., sulfur, antioxidant, an anti-caking agent (CPT), microcrystalline wax, accelerator, plasticizer, zinc oxide (ZnO), and stearate, were purchased from China Yangquan Olun Tape Co., Ltd. (Yangquan, China). All the reagents used were analytical grade, and the solutions were prepared using deionized water (DI).

### 2.2. Prescription

In units of phr (the weight fraction of rGO added to NR), and with the NR being 100, the following additives were used: 30 SBR, 10 carbon black, 9.2 antioxidant, 4.4 stearic acid, 3 microcrystalline wax, 3.0 ZnO, 2.9 stearate, 2.7 sulfur, 0.8 accelerator, 0.5 plasticizer, and 0.15 CPT. The other excipients totaled 16.8, and rGO was the variable, with a total of 176.35. The formula and vulcanization process were taken from an ST1600 (6 + 5 + 6)-type steel cord conveyor belt produced by China Yangquan Olun Tape Co., Ltd., Yangquan, China.

### 2.3. Equipment and Instruments

The following equipment and instruments were used: a two-roll mixer (ZC-DSRL-KL004B-150), Zhongcheng Precision (Dongguan, China); a mixing machine (WQ-1010), Weiqing Machinery (Jingjiang, China); a rotorless vulcanizer (GT-M2000-A), GOTECH (Qingdao, China); a plate vulcanizing machine (TY-7006), GOTECH (Qingdao, China); a pulling force test machine (AI-7000M), GOTECH (Qingdao, China); a DIN abrasion tester (GT-7012-DHT), GOTECH (Qingdao, China); a hardness tester (XY-1), GOTECH (Qingdao, China); an in situ Raman spectrometer (Invia Reflex, Cambridge, MA, USA), PANalytical, Malvern, UK; a scanning electron microscope (Gemini 300), Zeiss, Oberkochen, Germany; a hot air aging box (YF501-B), YuanFeng (Yangzhou, China); a thermal conductivity tester (DRL-III), XiangYi (Xiangtan, China); a multi-channel temperature recorder (TP1000), TOPRIE (Shanghai, China); and a thermocouple (TT-K-30), TOPRIE (Shanghai, China).

### 2.4. Preparation of rGO/NR Raw Rubber

**Figure 2:**
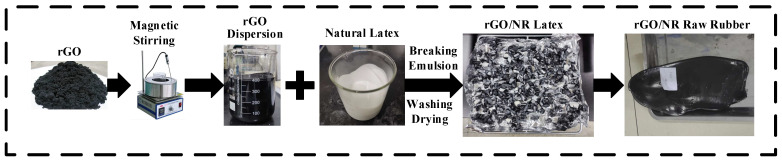
The preparation process of rGO/NR raw rubber.

The preparation process of the rGO/NR raw rubber is shown in Figure 2. rGO nanomaterials of varying quality (0~1.28 g) were fully mixed using mechanical stirring in DI to obtain an rGO dispersion (5 mg/mL) [17]. Then, the rGO dispersion was fully mixed with 71.33 g of natural latex, and calcium chloride flocculant with a mass fraction of 1 was slowly added to the mixed system to obtain an rGO/NR flocculation masterbatch. Finally, this was cut into pieces, washed, and dried in a vacuum oven at 50 °C for 24 h to obtain the rGO/NR raw rubber [18].

### 2.5. Preparation of rGO Rubber Cover Joint

**Figure 3:**
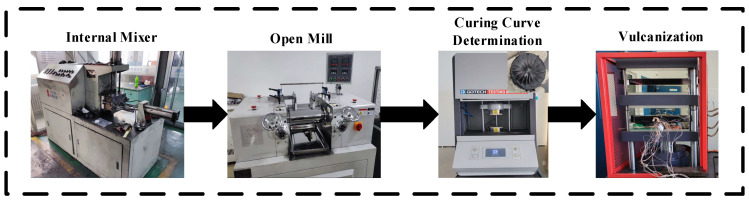
The preparation process of an rGO rubber cover joint.

The preparation process of the rGO rubber cover joint is shown in Figure 3. The temperature in the internal mixer was 110 °C, and the rotating speed was 40 r/min. rGO/NR raw rubber and SBR were added, in turn, for 4 min, and the other vulcanization auxiliaries were mixed for 8 min [19]. The temperature during two-roll mixing was 60 °C, and the rotating speed was 15 r/min. rGO raw rubber was rolled twice at a thickness of 2 mm, and sulfur was added at a thickness of 1 mm. The whole refining cycle was completed within 15–18 min, and the rGO rubber cover joint was obtained after 24 h at room temperature [20].

The rubber was rolled 6 times and triangle-wrapped 10 times; the thickness was adjusted by 2 mm, and the sheet was thin [21]. The whole rolling cycle was completed within 15–18 min, and the rGO composite raw rubber was obtained after standing for 24 h. rGO composite raw rubber was vulcanized in a flat vulcanizer. The curing temperature was 160 °C, the curing pressure was 2 MPa, and the curing time was TC90 + 5 min. The rGO rubber composite was thus obtained. TC90 was determined using a vulcanizer.

**Figure 4:**
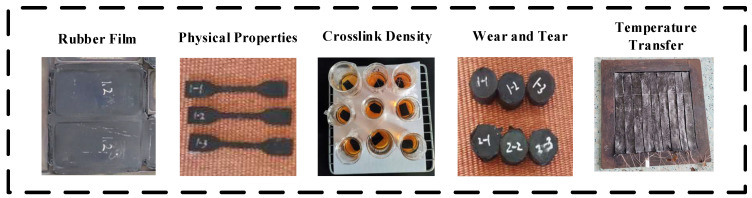
Samples of rGO/NR raw rubber with different shapes.

The rGO rubber joint cover is prepared into different shapes using molds according to test requirements before and after vulcanization. Various samples, such as film with a thickness of 2 mm, rubber pellets with a diameter of 10 mm, and dumbbell-shaped films, were prepared for various characterization tests, as shown in Figure 4.

### 2.6. Characterization

#### 2.6.1. XRD Analysis

X-ray diffraction (XRD) was used to test the crystal structure of the rGO/NR raw rubber composite [22]. The testing angle was 2θ, the range was 10~80, and the scanning rate was 10°/min.

#### 2.6.2. Raman Analysis

Defects in the rGO in the rubber composites were determined using an InVia Raman spectrometer [23]. The laser wavelength was 532 nm, and the range was 250–2500 cm^−1^.

#### 2.6.3. SEM Analysis

The dispersion of rGO in the rGO/NR raw rubber composites was observed using a scanning electron microscope (SEM) [24]. Before observation, an rGO rubber sheet with a thickness of 3 mm was subjected to liquid nitrogen low-temperature brittle fracture and gold-spraying treatments, and the fracture surface morphology was observed at 10 kV voltage.

#### 2.6.4. Mechanical Performance Testing

The characteristic curing parameters of the rGO rubber cover joint were tested with a rotorless vulcanizer. The rGO composite raw rubber was cut into circular rubber sheets with a diameter of 50 mm and a thickness of 4 mm and placed in the vulcanizer chamber for testing. The test temperature was set to 145 °C [25]. The tensile properties of the rGO rubber cover joint were tested with an electronic tensile machine according to the GBT528-2009 standard [26]. The tear strength was tested according to the GBT529-2008 standard [27]. A Shore A hardness test was conducted according to the GBT2411-2008 standard [28].

The cross-linking density of the rGO rubber composite was measured using the equilibrium swelling method [29]. After placing a 0.5 g sample in 30 mL toluene solution at 30 °C for 72 h, the sample was taken out and weighed, and the cross-linking density of the rGO in rubber composite was calculated according to the following formula:

$$V_{e}=\frac{\ln(1-V_{r})+V_{r}+\chi {V_{r}}^{2}}{V_{s}\left({V_{r}}^{\frac{1}{3}}-\frac{1}{2}V_{r}\right)}\;V_{r}=\frac{\left(m_{2}-m_{0}\varphi \right)\rho_{s}}{\left(m_{2}+m_{0}\varphi \right)\rho_{s}+\left(m_{1}+m_{2}\varphi \right)\rho_{r}}$$

where $V_{e}$ denotes the cross-linking density of rubber; $V_{r}$ represents the molar volume of toluene solution; χ signifies the solvent–rubber interaction parameter (the interaction parameter values of NR and SBR with toluene are 0.393 and 0.0653, respectively); *m*_0_ denotes the mass of rubber before swelling; *m*_1_ represents the mass of rubber after swelling; *m*_2_ signifies the mass of rubber after drying; φ  denotes the mass fraction of filler; and $\rho_{s}$ and $\rho_{r}$ represent the densities of the toluene solution and the NR/SBR, respectively.

According to GBT 1689-2014 standard [30], the wear resistance of the rGO rubber cover joint was tested with an Akron Abrasive Machine [31], and the angle difference between the sample and the grinding wheel was 15°.

#### 2.6.5. Adhesion Strength Testing

**Figure 5:**
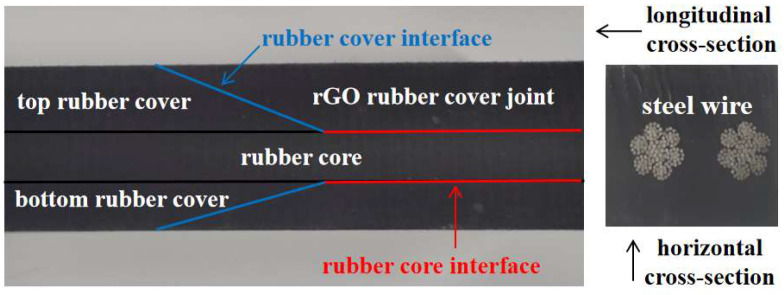
Wire rope conveyor belt joint structure diagram.

In order to detect the adhesion strength of the rGO rubber cover joint to the original conveyor belt rubber cover and rubber core, according to GB/T 30691-2014 [32], a test was carried out at a temperature of (23 ± 2) °C and a relative humidity of (50 ± 5)%. The structure of the joint of the steel wire conveyor belt is shown in Figure 5. First, the unvulcanized original rubber cover of the ST1600-800 (6 + 5 + 6)-type steel wire rope conveyor belt and 9 groups of rGO rubber cover joint with different addition amounts were processed into two 4 mm thick rubber sheets. The two rubber sheets were bonded up and down and vulcanized with a flat machine. The vulcanization conditions were consistent with the previous conditions. After standing for 5 days, the vulcanized film was cut into a 300 mm × 25 (±1) mm rectangular specimen with a thickness of 8 mm. The adhesive layer between the rGO rubber cover joint and the original rubber cover at the end of the specimen was peeled off approximately 75 mm. The specimens were then clamped separately at the ends of the rGO rubber cover joint and the original rubber cover using fixtures, and the rubber interlayer peeling test was carried out at a constant speed of (100 ± 10) mm/min with a tensile testing machine. According to the provisions of ISO6133 [33], the median peel force $\bar{F}$ was determined from the peeling force curve, and the adhesive strength between the rGO rubber cover joint and the core rubber was calculated by dividing the result by the width b (25 mm) of the rubber specimen, as seen in the formula below. Each test was conducted three times, and the results were averaged.

$$T=\frac{\bar{F}}{b}$$

Here, T denotes the adhesion strength of rubber reported in N/mm; $\bar{F}$ represents the median peeling force expresses in N; and b signifies the width of the pattern reported in mm.

The testing method for the adhesive strength of the rGO rubber cover joint and rubber core is consistent with the above.

#### 2.6.6. Thermal Conductivity Testing

The thermal conductivity of the rGO rubber cover joint was tested using a DRL-III thermal conductivity tester. Prior to testing, the samples were cut into cylindrical shapes with a diameter of 30 mm and a thickness of 20 mm. Standard specimens were used for thermal conductivity calibration before testing. During testing, it was necessary to promptly replace the mixture of ice and water. The temperature of the cold pole was set to 30 °C, while the hot pole temperature was set to 90 °C. Each test was conducted three times, and the average thermal conductivity of the rGO rubber cover joint was calculated.

#### 2.6.7. Thermal Aging Testing

According to the GBT 3512-2014 standard [34], thermal aging tests were conducted on the rGO rubber cover joint. The thermal air aging temperature was set to 75 °C. Dumbbell-shaped specimens of the rubber cover joint with nine different rGO addition levels were placed into the thermal air aging chamber, with ventilation occurring every hour. The thermal aging duration for the rGO rubber cover joint was 120 h, and specimens were removed every 24 h to measure their tensile strength.

#### 2.6.8. Conveyor Belt Vertical Temperature Conduction Testing

**Figure 6:**
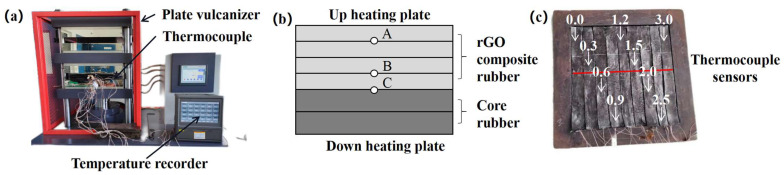
Vertical temperature test of the conveyor belt rubber: (**a**) test platform, (**b**) schematic diagram of thermocouple distribution in the vertical direction, and (**c**) curing mold.

In order to investigate the actual heat transfer performance of the rGO rubber cover joint in conveyor belts, a vertical temperature detection bed was built to test the rubber conveyor belt, as shown in Figure 6a, which included a plate vulcanization machine, a multi-channel temperature recorder, and a thermocouple temperature sensor. Referring to the dimensions of the ST 1600 steel wire rope conveyor belt, we produced the rubber layer above the steel wire. The production process is described as follows. The rGO raw rubber (covering rubber) and the prototype conveyor belt rubber core were processed into 3 mm and 6 mm thick films with a smelting machine and then cut into 40 × 360 rubber pieces.

Two layers of rubber core were added under the four layers of rGO raw rubber, and three thermocouple sensors (labeled as A, B, and C) were inserted between the rubber pieces in the same vertical plane, as shown in Figure 6b. In total, 9 groups of rubber with different rGO additions (0.0~3.0 phr) were placed in the mold, arranged from left to right. Thermocouple sensors were positioned at the red locations, as shown in Figure 6c, from left to right. Finally, the whole test object was put into the plate vulcanization machine for vulcanization, and the multi-channel temperature recorder measured the temperature everywhere in real time. The following experimental conditions were used: the temperature of the upper heating plate was 160 °C, the pressure was 2 MPa, and the acquisition interval was 0.1 s.

## 3. Results and Discussion

### 3.1. Characterization of the rGO/NR Rubber

**Figure 7:**
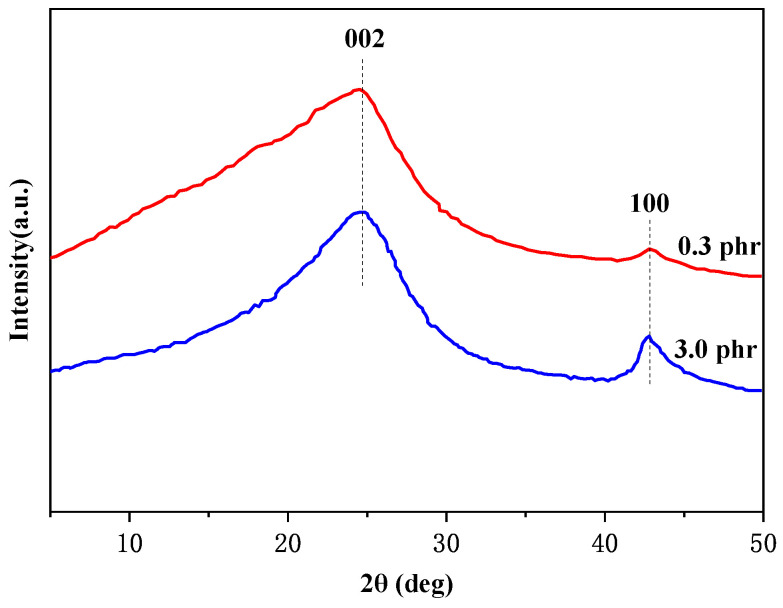
XRD patterns of rubber composites with different rGO additions.

The crystalline structures of various fillers were characterized using X-ray diffraction (XRD), and the diffraction patterns are shown in Figure 7. The rGO exhibited a broad diffraction peak at 2θ = 24.8°, corresponding to the 002 crystal plane, indicating a decrease in the interlayer spacing of the rGO layers. The diffraction peak at 2θ = 42.8° corresponded to the 100 crystal plane, suggesting the formation of stacked layer structures in the rGO [35]. When different amounts were added, the positions of the X-ray diffraction characteristic peaks of the rGO/NR raw rubber did not show significant shifts, indicating that the addition of rGO did not alter the crystalline structure of the rubber matrix. However, at 3.0 phr, the intensity of the diffraction peak at 2θ = 42.8° for rGO/NR latex increased, suggesting the possible aggregation behavior of the rGO layers.

**Figure 8:**
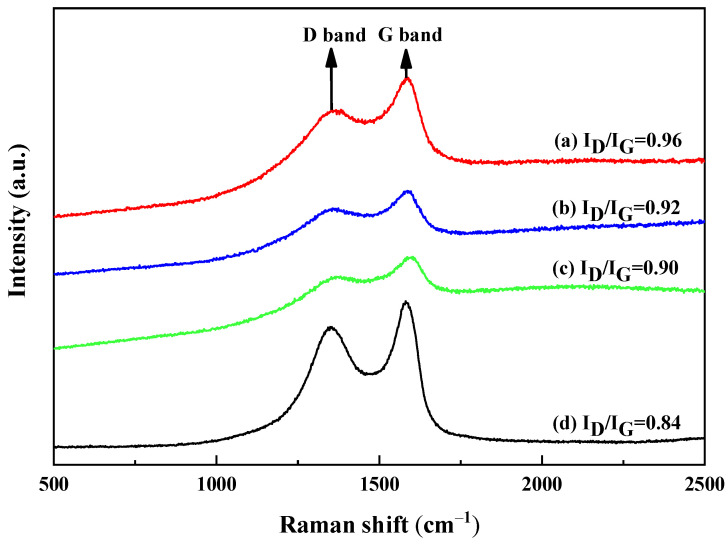
Raman spectra: (**a**) rGO with 3.0 phr, (**b**) rGO with 1.2 phr, (**c**) rGO with 0.3 phr, and (**d**) raw rGO.

To further investigate the change in the rGO structure, the Raman spectra for different fillers were analyzed, as shown in Figure 8. All the samples showed two characteristic peaks corresponding to the D (1362 cm^−1^) and G bands (1585 cm^−1^), which are related to the defects of the sp^3^ hybrid carbon or the disordered structure of the graphite and the in-plane vibration of the graphite lattice [36]. Compared with the raw rGO, the D and G bands of the rGO composites showed no obvious deviation, but the ratio of I_D_/I_G_ increased with the addition of rGO. These results indicate that the revived conjugation of rGO and the increasingly disordered structure resulted in defects, subsequently resulting in a deterioration in the bonding performance with the rubber matrix. As a result, it affected the mechanical properties and chemical stability of the conveyor belt.

### 3.2. Microstructure Analysis of the rGO Rubber Cover Joint

**Figure 9:**
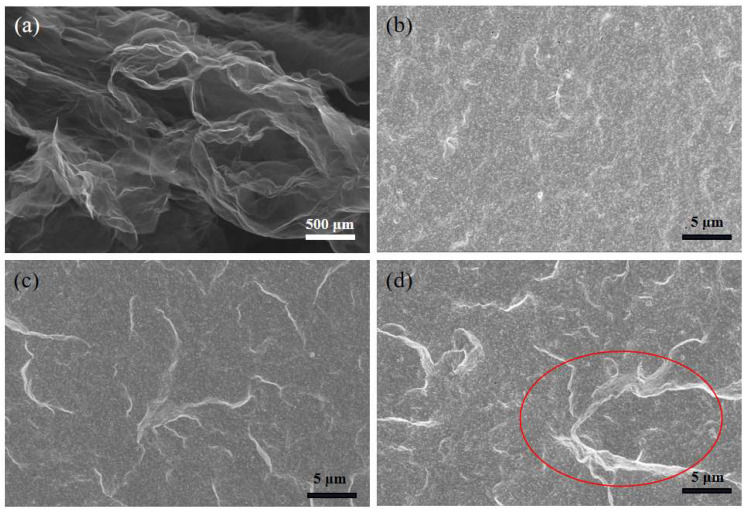
SEM images of (**a**) the raw rGO, (**b**) the rGO rubber cover joint (0.3 phr), (**c**) the rGO rubber cover joint (1.2 phr), and (**d**) the rGO rubber cover joint (3.0 phr).

As shown in Figure 9, the micro-morphology of the raw rGO and the rGO rubber cover joint was characterized using scanning electron microscopy (SEM). Figure 9a shows that the GO had a two-dimensional structure with multilayered platelets and a smooth flaky structure with abundant holes and a large specific surface area [37]. It can be seen from Figure 9b,c that the dispersion throughout the rubber matrix was better when a small amount of rGO was added. The rGO constructed a filler network in the matrix, which could bind the rubber molecular chain and improve the mechanical strength of the composite material. However, when the content was increased to 3.0 phr, as shown in Figure 9d, the rGO agglomerated in the rubber composites, which adversely affected the mechanical properties of the rubber composites.

### 3.3. Analysis of Vulcanization Characteristics

**Figure 10:**
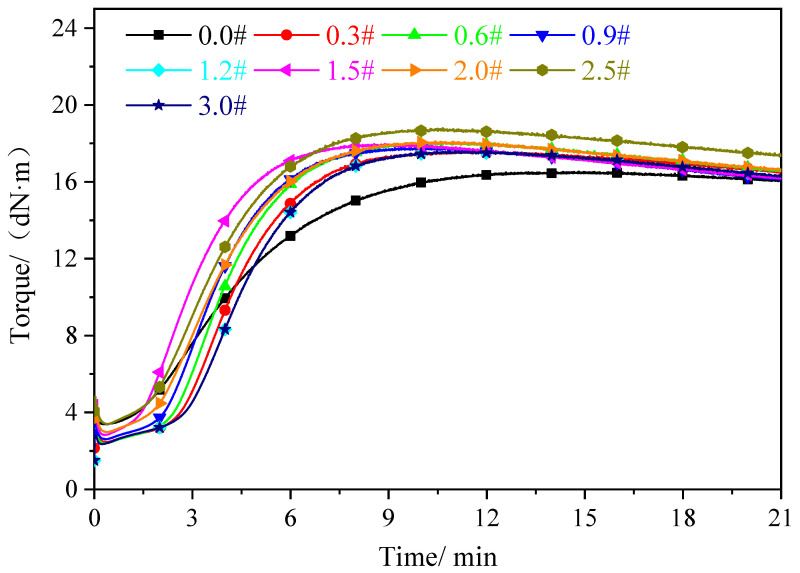
Vulcanization curve of the rGO rubber cover joint.

The vulcanization curves of the rGO rubber cover joint are shown in Figure 10. Compared with the original formula (curve 0.0#), the rubber composite with added rGO (curves 0.3#~3.0#) showed an increase in the rate of vulcanization, indicating that the rubber underwent vulcanization quickly. This is because the presence of oxygen functional groups in rGO accelerates the vulcanization of rubber composites.

The original rubber cover (0.0#) exhibited a slow increase in torque during the vulcanization process, completing vulcanization after 11 min, whereas the torque in the rubber cover with added rGO significantly increased, with vulcanization completion occurring at around 8 min. This indicates that the rubber rapidly underwent vulcanization. This is attributed to the oxygen functional groups (carboxyl and hydroxyl groups) in the rGO acting as catalysts to promote the vulcanization reaction, thereby facilitating cross-linking between the rubber chains and accelerating the vulcanization of the rubber cover joint. Additionally, due to the excellent thermal conductivity of the rGO, heat transfer was promoted during vulcanization, further inducing its faster occurrence. Consequently, during the induction period TC10, the vulcanization of the rGO rubber cover joint was prolonged. With the increase in the amount of rGO added, both the ML and MH of the rGO rubber cover joint were enhanced. These findings were attributed to the following: (1) the efficient and uniform dispersion of rGO throughout the rubber matrix and (2) the better interfacial compatibility between rGO and the rubber matrix. Therefore, under the dual effects of uniform dispersion and good interfacial interaction, the rGO better reinforced the rubber matrix.

Table 1 shows the vulcanization characteristic parameters of the rGO rubber cover joint. The maximum torque (MH) reflects the maximum cross-linking degree of the vulcanized rubber, and the minimum torque (ML) represents the interaction between fillers. MH–ML is often used to represent the equivalent cross-linking density of rubber compounds and the interaction between the matrix and the rGO. The analysis of the torque data shows that the addition of rGO can significantly increase the maximum torque of rubber composites. With the increase in the amount of rGO added, the torque difference of the composite material gradually increased. This was due to the strong interaction between the rGO and the rubber matrix, where the mesh structure surface of the rGO adsorbed the rubber macromolecules, leading to an increase in the amount of filler and the formation of a filler network [38]. T_C10_ and T_C90_ gradually extended with the increase in rGO dosage, which is mainly due to the adsorption effect of the oxygen-containing functional groups on the rGO surface of the accelerators and vulcanizing agents, which can prevent the rubber from being easily scorched and improve the safety of conveyor belt processing.

### 3.4. Cross-Linking Density

**Figure 11:**
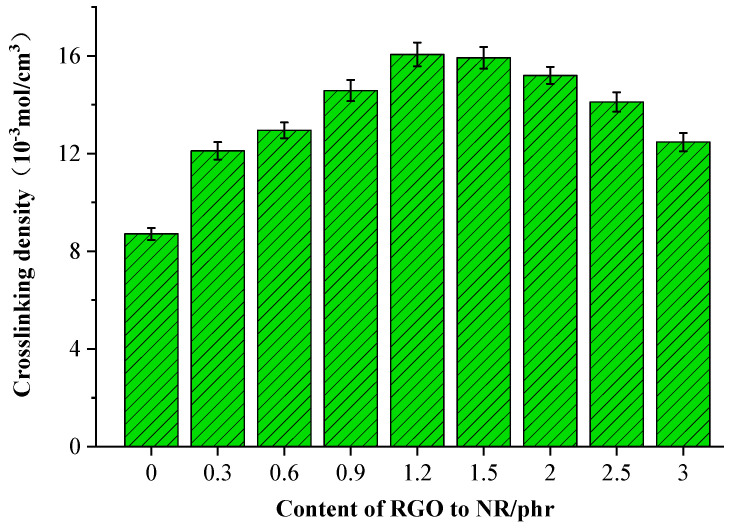
Cross-linking densities of the rGO rubber cover joint.

Figure 11 presents the cross-linking densities of the rubber cover joint with different rGO additions. It is evident that the cross-linking density of the rubber cover joint exhibits a trend of initially increasing and then decreasing. The addition of rGO enables physical adsorption with rubber molecules due to its surface properties, thereby enhancing the connections between them and leading to an increase in the cross-linking density of the rubber material, making it denser and more robust. However, at high addition levels, the cross-linking density of the rubber cover joint decreases, which is consistent with the trend in tensile strength, with both attributed to the agglomeration of rGO. Aggregation of rGO results in several layers clustering together, reducing the total specific surface area of the filler, leading to a decrease in the number of physical bonding points formed within the rubber and thus causing a decline in cross-linking density. When the rGO addition level reaches 1.2 phr, the cross-linking density reaches its maximum, with an increase of 80.6% compared to the original rubber cover formula. The error of the three tests was within 5%.

### 3.5. Physical Characteristics

**Figure 12:**
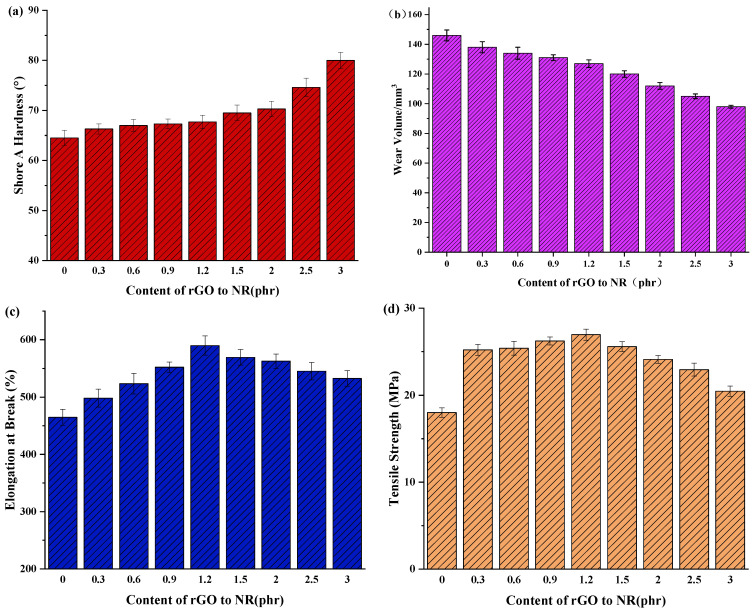
Physical properties of the rGO rubber cover joint. (**a**) Shore A hardness, (**b**) Wear volume, (**c**) Elongation at break, (**d**) Tensile strength.

The physical properties of the rGO rubber cover joint are shown in Figure 12. The Shore A hardness of the rGO rubber cover joint is shown in Figure 12a. The hardness of the rubber cover joint increased continuously with the increase in rGO. The original rubber cover had a hardness of 64.5. As more rGO was added, the hardness of the joint rubber cover gradually increased. This is attributed to the excellent mechanical strength and rigidity of the rGO. The carbon–carbon bonds and the two-dimensional lattice structure of the rGO effectively disperse and transmit stress, preventing the slippage and fracture of rubber molecules. Consequently, this enhances the hardness of the rubber, improving the wear resistance and compressive strength of the conveyor belt, thereby extending its service life. When the rGO content is 1.2 phr, the hardness reaches 67.7, representing a 5% increase. In previous analysis, it was determined that when the rGO addition exceeds 1.2 phr, agglomeration may occur. Nevertheless, even in this scenario, the agglomerated rGO can still fill the gaps between rubber molecules, providing a filler effect, thereby increasing the hardness of the rubber. However, it is worth noting that an excessively hard rubber cover can lead to negative effects, such as the reduced elasticity of the conveyor belt, decreased bending capability, and weakened tensile strength. This can increase the risk of wear and damage.

The wear volume of the rGO joint rubber cover is shown in Figure 12b. It can be seen that with the increase in the rGO content, the wear loss of the rubber composites decreased, and the wear resistance increased. When the contents of rGO totaled 1.2 phr and 3.0 phr, the wear loss was reduced by 13.0% and 32.9%, respectively, compared with that of the original formula. This was mainly due to the strong interaction between functional groups on the surface of rGO and the rubber molecular chain [39], which limited the movement of rubber molecules and reduced the deformation ability of the friction surface, resulting in a decrease in the area of wear.

Figure 12c,d show the elongation at break and tensile strength of the rubber cover with different rGO additions. Compared with the original formula, the mechanical properties of the rubber composites with a small amount of rGO were greatly improved. The elongation at break and tensile strength increased by 26.9% and 49.7%, respectively, when the dosage was 1.2 phr. This finding was due to the following features: (1) rGO has a two-dimensional lamellar structure and high specific surface area, which forms an overlapping filler network, enhancing the interface interaction between the rGO and the rubber matrix as well as improving the tensile strength; (2) the lamellar filler network has a strong limiting effect on the rubber molecular chain, resulting in a decrease in the elongation at break of the rubber composites; and (3) the latex blending method ensures that rGO is evenly dispersed throughout the rubber matrix [40,41]. However, with the continuous increase in the rGO, the tensile strength of the rubber composites decreased sharply, and the elongation at break increased. This may have been due to the agglomeration of excessive rGO in the rubber composites, leading to the stress concentration of materials or strong interaction between rGO and the rubber composites, limiting the slippage of polymer chains.

### 3.6. Adhesion Properties

**Figure 13:**
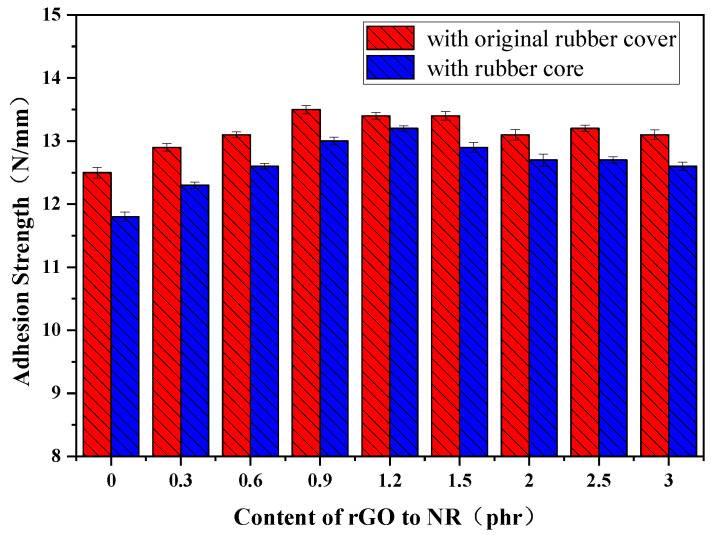
Adhesion strength of the rGO rubber cover joint.

Figure 13 illustrates the adhesion strength between the rubber cover joint and the original rubber cover, as well as the rubber core, when different amounts of rGO are added. The adhesion strength between the vulcanized original rubber cover (0#) was 12.5 N/mm. With the addition of rGO, the adhesion strength between the rubber cover joint and the original rubber cover slightly increased. When the rGO content ranged from 0.9 to 1.5 phr, the adhesion strength of the rubber cover joint reached a relatively good state. This is attributed to the dependency of adhesion strength between rubbers on intermolecular forces. The added rGO contains hydroxyl (–OH) and carboxyl (–COOH) chemical bonds, with the carboxyl groups exhibiting strong electrophilicity. These groups undergo esterification reactions with the carbon–carbon double bonds (–C=C) on the rubber surface, forming stronger and more stable covalent bonds, thereby enhancing the intermolecular forces at the bonding interface and resulting in a more robust connection. As the rGO content increases, the adhesion strength of the rubber cover joint gradually decreases. This may be attributed to the reduction in the contact area between filler particles when rGO aggregates, leading to a weakening of interfacial interactions and consequently weaker adhesion strength. Additionally, it is possible that the uneven distribution of filler particles in the rubber matrix due to high rGO content results in localized weakening, further reducing the adhesion strength. When the rGO content ranges from 0.9 to 1.5 phr, the adhesion strength of the rubber cover joint remains at a level close to that, with the adhesion strength at 1.2 phr approximately 13.4 N/mm, representing a 12.4% enhancement.

The trend of the adhesion strength variation between the rGO rubber cover joint and the rubber core is consistent with that of the original rubber cover, but the strength values are slightly lower. When 1.2 phr was added, the adhesion strength was 13.2 N/mm, which is a 12.2% increase. This is because the formulation of the rGO rubber cover joint is similar to that of the original rubber cover, ensuring good compatibility.

### 3.7. Thermal Conductivity

**Figure 14:**
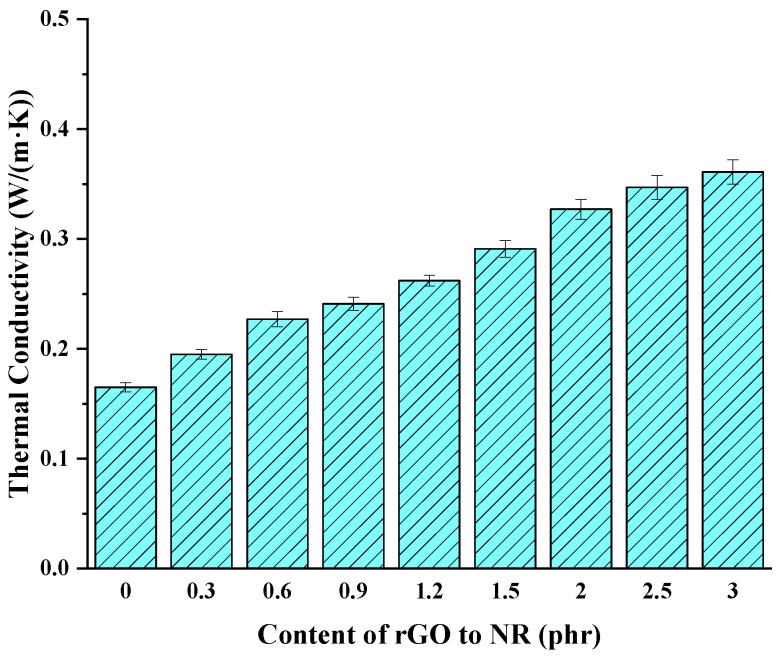
Thermal conductivity of the rGO rubber cover joint.

To investigate the influence of rGO on the thermal conductivity of the joint rubber during conveyor belt operation, the thermal conductivity of fully vulcanized rGO rubber cover joint was tested, as shown in Figure 14. It can be observed that with increasing rGO addition, the thermal conductivity of the rubber cover joint gradually increases. This is attributed to the excellent thermal conductivity property of rGO itself. When rGO is added to rubber, it can physically adsorb onto the surface of rubber molecules, leading to tighter and more organized connections between rubber molecules, thereby facilitating smoother heat conduction within the rubber. Additionally, better interface contact between rGO and the rubber matrix reduces interfacial thermal resistance. However, at high addition levels, rGO fillers tend to aggregate, forming clusters or agglomerates. This reduces filler dispersion and affects thermal conduction efficiency, leading to a decrease in the rate of thermal conductivity enhancement. When the addition level is 3.0 phr, the thermal conductivity of the rGO rubber cover joint is increased by 141.2% compared with the original rubber cover.

### 3.8. Thermal Aging

**Figure 15:**
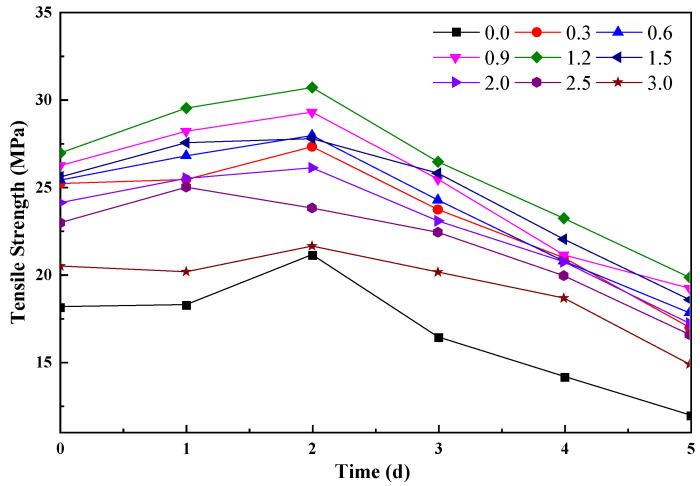
The impact of rGO addition on the thermal aging performance of the rubber cover joint.

Figure 15 illustrates the impact of rGO addition on the thermal aging performance of the rubber cover joint. In the early stages of thermal aging, the tensile strength of the rGO rubber cover joint gradually increases. This is due to the release of residual stresses within the rubber during the preparation process, while the rubber material continues to undergo cross-linking reactions, forming a stronger molecular network structure. However, over time, the tensile strength of the rubber material sharply decreases. This is because polymer chains undergo degradation reactions at high temperatures, leading to the breakage of rubber molecular chains. After 5 days of thermal aging, the tensile strength of the original formula rubber is only 65.7% of that before the process. The addition of rGO enhances the overall tensile strength of the rubber material at various addition levels. This is attributed to the excellent thermal conductivity of rGO, as demonstrated in the previous section. When added to rubber, rGO forms a thermal conductivity network, facilitating faster heat conduction. Additionally, added rGO promotes cross-linking between rubber molecules, forming a more stable network structure that enhances the overall stability of the rubber. When the addition level is 1.2 phr, the tensile strength of the rubber cover joint can still be maintained at 72.5% of the pre-thermal aging level, demonstrating the positive role of rGO in improving the aging resistance of rubber materials.

### 3.9. Actual Thermal Conductivity Performance of rGO on Conveyor Belts

**Figure 16:**
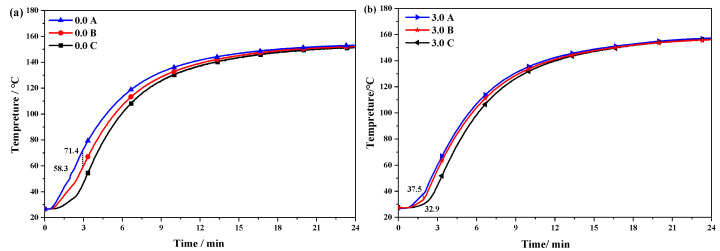
Heating curve of the rGO rubber in the vertical direction: (**a**) 0.0 rGO and (**b**) 3.0 rGO.

The curing temperature curve of the rGO rubber composite is shown in Figure 16. From Figure 16a, it can be seen that the heat generated by the vulcanizer is conducted inward from the original formula rubber cover, resulting in a significant temperature difference between positions A and B, and the maximum temperature difference in the early heating stage reached 13.1 °C. Since conveyor belt rubber is a low-thermal-conductivity material, temperature differences lead to different curing levels at each vertical position, especially when the central rubber is not fully vulcanized and the external rubber is over-vulcanized, which, in turn, leads to a reduction in the conveyor belt strength [42,43].

Figure 16b shows the temperature curve of the rubber in the vertical direction with 3.0 phr rGO. It can be seen that the temperature difference between A and B was greatly reduced; the maximum temperature difference was only 4.6 °C. This was because the interface thermal resistance was reduced due to the combination of rGO with high thermal conductivity and the rubber matrix; a good thermal conduction path was established, thus improving the thermal conductivity of the composites [44].

In order to further explore the influence of the rGO addition on the thermal conductivity of the rubber composites, the temperatures of the rubber composites with different rGO additions at the end of vulcanization were analyzed. As shown in Table 2, as more rGO was added, the temperature difference (Δt_AC_) between the inside and outside of the rubber continuously decreased, and the rate of decrease gradually slowed down. This is consistent with the test results in Section 3.7, where the addition of rGO enhances the thermal conductivity of the rubber cover of the joint. During the vulcanization process of the steel wire rope conveyor belt joint, reducing the temperature difference between the inside and outside of the rubber improves the vulcanization effect. Specifically, when 1.2 phr and 3.0 phr rGO were added, the temperature difference between points A and C decreased from 4.5 °C to 3.3 and 1.8 °C, respectively.

## 4. Conclusions

In summary, utilizing the established formula of a commercially available steel wire rope conveyor belt, we fabricated an rGO rubber cover joint using latex mixing and mechanical blending techniques. Our investigation focused on assessing how varying amounts of rGO affect the characteristics of rubber cover composites. Additionally, we conducted conveyor belt vertical temperature conduction testing to analyze the thermal conduction performance of the rGO cover joint in practical applications. The main conclusions are as follows:

(1) The rGO dispersed uniformly in the rubber composites at a low dosage, but when 3.0 phr rGO was added, it agglomerated in the rubber composite.
(2) The analysis of vulcanization characteristics showed that adding rGO can prolong the curing scorch period of a rubber compound and thus improve the safety of the rubber vulcanization process. With the increase in the rGO content, the mechanical properties of the rubber composites first improved and then worsened. When 1.2 phr was added, the mechanical properties of the rubber were highest. Compared with the original formula, the cross-linking density increased by 80.6%, the tensile strength increased by 49.7%, the elongation at break increased by 23.6%, and the adhesion strength between the rGO rubber cover joint and the original rubber cover, as well as the rubber core, increased by approximately 12.4%. The tensile strength of the rGO rubber cover joint still maintained 72.5% of its pre-thermal aging value. In addition, the wear resistance and thermal conductivity of the rubber increased as more rGO was added. When 3.0 phr rGO was added, the wear resistance of the rubber improved by 32.9%, and the thermal conductivity increased by 118.8%.
(3) The thermal conduction test of the rGO rubber cover joint on the conveyor belt shows that the addition of rGO can improve the uniformity of the internal and external temperatures of rubber during vulcanization. The temperature difference was reduced from 4.5 °C to 1.8 °C, improving the vulcanization quality. Therefore, this work provides important information regarding the industrial production of high-strength steel wire rope core conveyor belts.

## Figures and Tables

**Table 1:** Vulcanization characteristic parameters of the rGO rubber cover joint.

Sample	MH	ML	T_C10_/min	T_C90_/min	MH-ML	T_C90_-T_C10_/min
0.0 ^#^	14.70	2.74	01:30	04:07	13.76	2:37
0.3 ^#^	17.56	1.88	01:37	04:19	15.68	2:42
0.6 ^#^	17.31	1.18	01:43	04:40	13.13	2:57
0.9 ^#^	17.76	1.98	01:47	04:54	15.78	3:07
1.2 ^#^	17.54	1.69	02:03	05:15	15.85	3:12
1.5 ^#^	17.91	2.16	02:08	05:25	15.75	3:17
2.0 ^#^	18.04	2.31	02:21	05:55	15.73	3:34
2.5 ^#^	18.72	2.67	02:30	06:21	16.05	3:51
3.0 ^#^	19.16	4.07	02:49	06:45	16.12	3:56

^#^ represents the amount of rGO added to rubber (phr).

**Table 2:** Temperatures of each point at the completion of vulcanization.

Sample	0 #	0.3 #	0.6 #	0.9 #	1.2 #	1.5 #	2.0 #	2.5 #	3.0 #
t_A_/°C	154.4	154.3	154.2	154.3	153.9	153.9	154.0	153.7	153.5
t_B_/°C	150.7	150.9	151.2	151.2	151.4	15.1.3	152.0	152.4	152.9
t_C_/°C	149.9	149.9	150.3	151.0	150.6	151.2	151.4	151.7	151.7
Δt_AC_/°C	4.5	4.4	3.9	3.3	3.3	2.7	2.6	2.0	1.8

^#^ represents the amount of rGO added to rubber (phr).

## Data Availability

Data are contained within the article.
